# Investigation of the Impact of Single and Double Filtration Systems on Post-Consumer PE Film Waste

**DOI:** 10.3390/polym16162238

**Published:** 2024-08-06

**Authors:** Johanna Langwieser, Joerg Fischer

**Affiliations:** 1 Institute of Polymeric Materials and Testing, Johannes Kepler University Linz, Altenberger Strasse 69, 4040 Linz, Austria; 2 Competence Center CHASE GmbH, Altenberger Strasse 69, 4040 Linz, Austria

**Keywords:** plastic film waste, melt filtration, post-consumer recyclate, industrial trials, mechanical recycling

## Abstract

Due to the diversity of plastic film waste streams available on the market and the associated variety of contaminants’ size and number, the use of melt filtration is necessary. Currently, single and double filtration systems are state of the art in the plastic recycling industry, depending on the application of the produced post-consumer recyclate (PCR). Using PCR for thin films demands small contamination sizes, which are easier to reach using a second filtration step. In the case of relatively clean post-consumer input materials, it must be investigated whether the additional load from the second filter has a counterproductive effect on the material and whether single filtration would be sufficient. For this paper, polyethylene (PE) film waste stemming from a separate post-consumer collection in Austria was processed using an industrial-sized recycling machine with different combinations of filter sizes and systems. Melt flow rate (MFR), ash content, oxidation onset temperature (OOT), and optical contaminant detection were measured to investigate the influence of single and double filtration systems. The investigation showed that, even though the contamination amount and size were reduced, the second filter had a distinct effect on specific properties.

## 1. Introduction

Plastic packaging ensures the freshness of food, the safety of medicines, and the durability of fragile goods, providing convenience and reliability in our daily lives. In the European Union (EU), plastic converters have accounted for approximately 40% of the total plastic demand for packaging in recent years, which equates to approximately 20 million tons of plastic [1]. Among plastic packaging materials, the importance of flexible packaging cannot be overstated. When examining the global scenario for plastic packaging demand in 2019, flexible plastics accounted for approximately 58% of the total [2].

While flexible packaging has grown in popularity due to its many benefits, it is not without its drawbacks [3,4,5]. Widespread littering leads to negative environmental impacts such as wildlife deaths, pollution, clogging of sewers and waterways, and alteration of landscapes. Of particular concern is the long decomposition time of polymeric materials, which poses a significant environmental challenge [6,7]. To address these drawbacks, the European Union (EU) has implemented a directive [8] that aims to achieve specific recycling targets for plastic packaging, with a target of 50% by the end of 2025, and increasing to 55% by the end of 2030. Europe is currently transitioning from a linear economy to a circular economy model. The recycling rate of post-consumer plastic waste in Europe is currently around 26.9%. According to the new calculation methodology, recycling starts with the materials entering the pelletizing, extrusion, and molding processes, after the removal of impurities and unsuitable substances from the sorted materials [9]. Because sorting facilities work cost effectively, the quality of the less valuable streams is often compromised [10].

Beyond environmental concerns, plastic packaging film waste is considered a challenging recycling stream [11]. A number of technical barriers complicate the mechanical recycling of films and flexible packaging, including inefficiencies in collection and sorting, high levels of input contamination, low bulk density and therefore poor processing capabilities, and a lack of end markets [12]. As a result, flexible film waste is still often not recycled.

Many studies have looked at the environmental benefits of recycling. Since plastic film is still very often not recycled, studies such as that by Hou et al. [13] have shown that recycling has a better environmental impact than other end-of-life treatments. They determined the life cycle environmental impacts of recycling, landfill disposal, and incineration. In their study, they discovered that recycling appears to be ecologically more beneficial when the plastic film waste is recovered from mixed waste rather than waste from a separate collection due to the higher mass fraction of plastic film that exists in mixed waste despite the lower recycling rate. In addition, recycling has a greater environmental benefit than landfilling and incineration.

In terms of recycling, flexible film packaging can be recycled by either mechanical recycling or chemical (feedstock) recycling. Mechanical recycling preserves materials or macromolecules, whereas chemical recycling destroys materials and breaks down macromolecules into monomers or oligomers that can then be reused [14]. Perugini et al. [15] conducted a life cycle assessment of mechanical and feedstock recycling options for the management of plastic packaging waste (not limited to flexible film waste). Five plastic waste management options were evaluated. The focus was on mechanical recycling, which is close to the current plastic packaging management policy applied in Italy, compared to alternatives such as landfill, incineration, and mechanical recycling combined with low-temperature pyrolysis and combined with hydrocracking. The authors concluded that mechanical recycling is always environmentally preferable. However, feedstock recycling, such as the hydrocracking process, also represents an end-of-life scenario that avoids environmental burdens when compared with virgin materials. Nevertheless, mechanical recycling has some limitations in its application. Due to inherent contamination and regulatory concerns (e.g., food contact or medical appliances), chemical recycling can be a way to still keep the used material in the value chain.

In this study, mechanical recycling was performed. A conventional mechanical recycling process may include steps such as separation and sorting based on shape, density, size, color, or chemical composition, washing to remove contaminants, grinding to reduce products to flakes, compounding, and pelletizing, optionally with melt filtration [16]. Melt filtration removes non-melted contaminants from the melt, improving the quality and properties of the recyclate. Furthermore, melt filtration also improves the process stability in later processing stages [17].

Depending on the type and extent of contamination and the required product quality, single-stage filtration may be sufficient for certain applications [18,19,20,21,22,23]. In the case of high-quality recyclates from post-consumer film waste, studies have shown that a second melt filtration system improves the properties compared to a single filtration system [24].

However, the properties of virgin material cannot be achieved with recyclates. The mechanical recycling process itself affects certain properties of polyethylene (PE) by initiating degradation processes [25,26]. Contamination complicates the recycling process as each step affects the material. However, it is necessary to remove contaminants to improve, e.g., optical properties. Additionally, contaminants from multilayer products (e.g., polyamide) can degrade into other products, potentially leading to a problematic odor [27].

To put this paper in context, other studies have been reviewed and summarized in the following paragraphs. For flexible film recycling, the review written by Horodytska et al. [28] summarizes the current issues very well. Plastic film recycling rates are still very low. In addition, they identified a lack of research on the behavior of flexible films during different recycling processes. Multilayer products continue to be a major problem in recycling, and more efficient sorting technologies, compatibilization, and delamination processes should be investigated to make recycling technically and economically feasible. They recommend further research into closed-loop recycling systems and more research into the topic of deinking processes and other decontamination technologies. In addition, they emphasize the importance of life cycle assessment to evaluate the impacts on the environment.

Often, recycling is performed on a laboratory scale [29,30,31,32]. Few studies have investigated the impact of industrial-scale processing. Soto et al. [33] conducted a real case study of mechanical recycling of PE plastic films from mixed solid waste on the industrial scale. The main difference between this study and other recycling processes was the raw material used. The plastic films came from municipal solid waste, which required a prior separation step. The material was processed by shredding and sieving, pre-washing, vigorous washing, separation by hydrocyclones and magnets, thermal drying, extrusion, and pelletizing. The authors found that there was no significant effect of washing temperature on the parameters of the water. The results showed good properties, close to those of virgin PE.

So far, only one research group has investigated the effect of double filtration at an industrial size using post-consumer flexible waste. Bashirgonbadi et al. [24] investigated the quality and economic evaluation of a mechanical recycling process for post-consumer flexible plastics recommended by Ceflex (a collaborative initiative of a European consortium of companies that represents the entire value chain of flexible packaging) [34]. This recommended recycling process includes additional sorting, hot washing, and improved extrusion including a second filtration step. Overall, the granulates they studied resulted in greater flexibility, higher ductility, and improved tensile strength compared to conventional mechanical recycling processes.

In addition to the properties of the recyclates produced by the Ceflex process, Lase et al. [11] investigated the recycling performance of a conventional recycling process and the Ceflex recycling process. Based on a material flow analysis, a mathematical model was developed and applied. The results showed that the process yield of the Ceflex process is similar to that of the conventional recycling process. However, better polymer grades can be obtained, which may lead to a larger market segment of recycled flexible materials.

To further investigate the effect of additional filtration, industrial trials of single and double filtration systems were conducted using post-consumer polyethylene (PE) film waste to evaluate the effect on material properties and the difference between single and double filtration.

## 2. Materials and Methods

### 2.1. Investigated Materials

**Figure 1:**
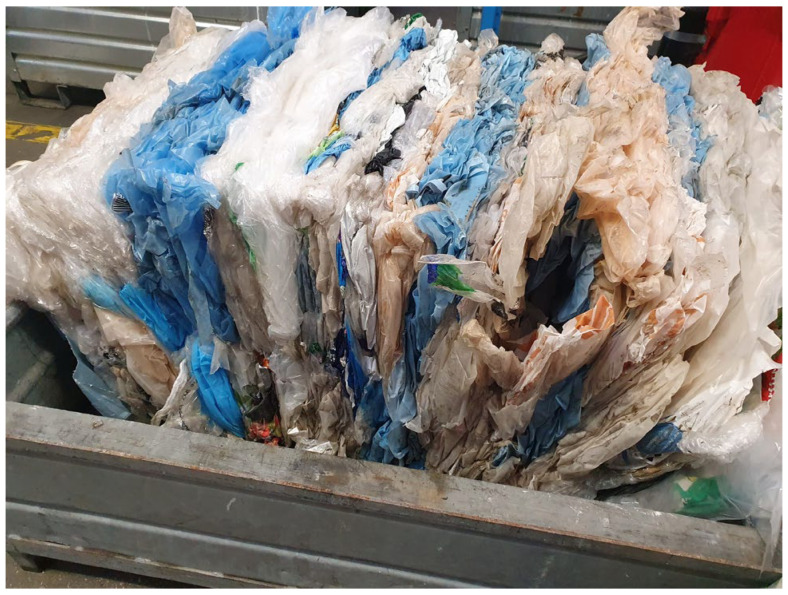
Post-consumer film waste collected in Upper Austria.

The investigated materials were obtained from a post-consumer waste collection center located in Upper Austria. Those waste collection centers are discrete collection points and facilitate the separate collection of up to 80 distinct materials and substances and are under the administration of Oberösterreichisches Landes-Abfallverwertungsunternehmen GmbH (LAVU) (Wels, Austria). The material used was the polyethylene (PE) packaging film waste fraction, which includes soft films, wrapping films, stretch films, carrier bags, small bags, and bubble wrap. To ensure a stable process, approximately four tons of this material was used. A picture of the films before pre-treatment can be found in Figure 1.

### 2.2. Pre-Treatment of the Film Waste

To be able to process the post-consumer PE packaging film fraction, certain pre-treatment steps are required to ensure a homogenized size of the waste films and a rather clean stream. As a first step, the material was cut to smaller sizes of approximately 40 to 60 mm using a Lindner Shredder Universo 2800 (Lindner-Recyclingtech GmbH, Spittal/Drau, Austria). Furthermore, the material was separated from higher-density materials and washed using a swim–sink tank with water at room temperature and anti-foaming agent (producer unknown) and further separated using a hydro cyclone (Herbold Meckesheim GmbH, Meckesheim, Germany). After the separation and washing step, the film flakes were dried using a friction washer (Herbold Meckesheim GmbH, Meckesheim, Germany). The material was then transported into a silo storage where further thermal heating was applied to achieve the desired residual moisture level of below 10%.

### 2.3. Single and Double Filtration Systems in the Recycling Extrusion Step

**Figure 2:**
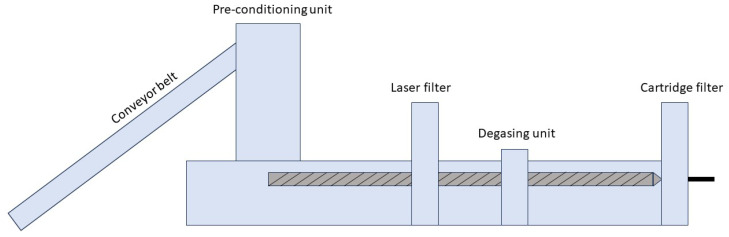
Schematic setup of the used industrial-sized recycling machine INTAREMA 1714 TVEplus (EREMA Engineering Recycling Maschinen und Anlagen Ges.m.b.H., Ansfelden, Austria).

After the pre-treatment, the material was directly processed using an industrial-sized recycling machine INTAREMA 1714 TVEplus (EREMA Engineering Recycling Maschinen und Anlagen Ges.m.b.H., Ansfelden, Austria). This machine has an average throughput capacity of PE of 1250 to 1550 kg/h; further information can be found in the technical data sheet [35]. The setup of the machine is shown in Figure 2. The melt is first filtered through a laser filter (LF), then degassed by a vacuum degassing unit and filtered a second time through a cartridge filter (CF). The LF was operated at a constant rotational speed of 10 rpm. The filter sizes and combinations are listed in Table 1. X in column CF indicates the single filtration step. Samples were collected from six different granulates produced. The temperature profile can be seen in Table 2. The opening from the pre-conditioning unit to the feed zone was opened 50% of its full capacity and the extruder processed with a screw speed of approximately 60 rpm.

### 2.4. Characterization Methods

The six differently filtered granulate samples were characterized by melt flow rate (MFR), ash content, oxidation temperature, and optical contamination detection. The MFR measurements were performed using the Aflow (ZwickRoell GmbH and Co. KG, Ulm, Germany) according to ISO 1133 [36] with a test weight of 2.16 kg and a measurement temperature of 190 °C. The preheated cylinder of the Aflow was filled with approximately 3 g of the material. It was further compacted and heated for five minutes. The measurement started automatically, and the piston pressed the molten material through the die. Six samples were cut from the middle strand and weighed. Each of the six samples was weighed twice.

The ash content was measured using the standard ISO 3451-1 [37]. Quartz fiber crucibles with a Phoenix microwave muffle furnace (CEM Corporation, Matthews, NC, USA) were used for the ashing process. Approximately 3 g of the material was placed into the crucibles and directly calcined in the muffle furnace at 750 °C for 15 min. The crucibles were weighed, and the ash content was calculated using Equation (1):

(1) $$A\%=\frac{m_{1}}{m_{0}}\times 100$$

where *A*% is the resulting ash content, *m*_0_ is the initial mass of the test sample, and *m*_1_ is the measured mass of the obtained ash. Each of the six samples was tested three times. Additionally, the input material (flakes) was calcined to make a comparison between input and output possible.

Measurements for the oxidation onset temperature (OOT) were performed using a DSC 4000 (Perkin Elmer Inc., Waltham, MA, USA). The heating rate was 10 K/min from 30 °C to 230 °C, and the mass of the specimens was 5.0 ± 0.5 mg. The samples were continuously heated in an air atmosphere until a baseline shift occurred at a characteristic temperature, indicating the onset of an exothermic event and thus oxidative degradation.

The optical contamination detection was performed using a Measuring Extruder ME30 and Modular Film Analyzer FSA100 (OCS Optical Control Systems GmbH, Witten, Germany). The extruder was set at a temperature of 30 °C in the feed zone, 180 °C in zone 1, 185 °C in zone 2, 190 °C in zone 3, 195 °C in zone 4, 200 °C in zone 5, and 200 °C in zone 6. The machine was run at a screw speed of 18 rpm, and a sheet extrusion die with a slit geometry of 150 mm × 1 mm was used. The cooling rollers were set at a temperature of 30 °C with a speed of 5.6 rpm. The film was kept at a tension of 6 N and wound up with a force of 7 N. The contamination measurements were performed at a base gray-scale value of 180. If a deviation of 20% (darker) occurred, the measuring system was triggered and recorded a contamination. The contaminations were categorized in sizes between 25 and 100 µm, 100 and 110 µm, 110 and 130 µm, 130 and 200 µm, and above 200 µm. Per sample, a total area of 10 m^2^ with a thickness of 50 µm was examined. The results were then normalized to 1/m^2^.

## 3. Results and Discussion

**Figure 3:**
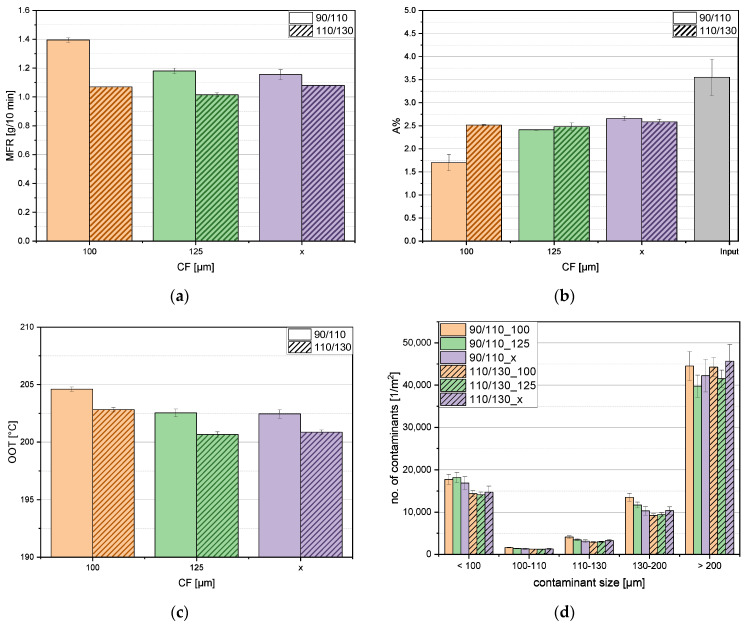
Results of the single and double filtration steps for (**a**) melt flow rate (MFR), (**b**) ash content (A%), (**c**) oxidation onset temperature (OOT), and (**d**) contaminant number and size.

**Figure 4:**
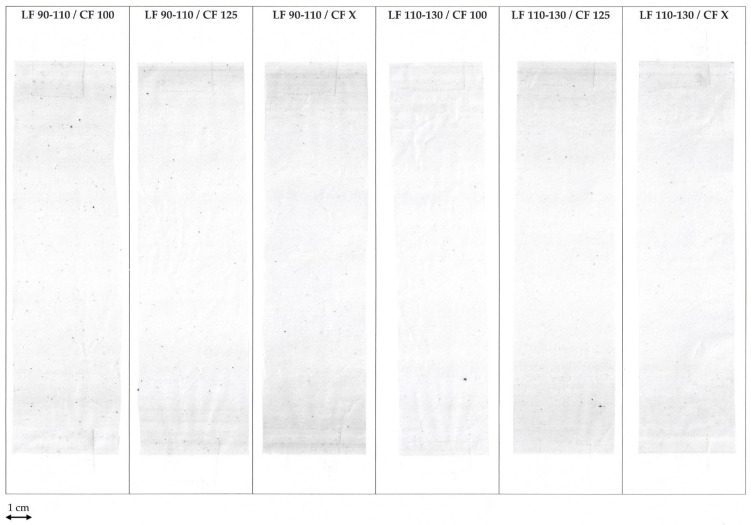
Picture of the produced OCD films.

The diagrams in Figure 3 are systematically color-coded for clarity. The clear columns represent outcomes associated with the smaller laser filter (LF) size (90/110 µm), whereas the striped columns depict results related to the larger laser filter size (110/130 µm). To distinguish between the cartridge filter (CF) sizes, distinct colors have been assigned; orange signifies 100 µm, green represents 125 µm, and violet corresponds to the single filtration step. Additionally, the input material was measured to determine the ash content. In this case, the column is colored gray. Figure 4 shows the six different OCD films. There are no clear differences between the films in this figure, so OCD measurements were performed to look more closely at the contaminations.

Figure 3a illustrates the melt flow rate (MFR) values, revealing distinct trends based on LF and CF sizes. The smaller LF size (90/110 µm) exhibits a consistent decrease in the MFR with increasing filter size. Specifically, material filtered through the smaller CF size of 100 µm attains an MFR of 1.4 g/10 min, while the CF with 125 µm and the single filtration step achieve approximately 1.2 g/10 min and 1.15 g/10 min, respectively. Notably, the combination of the smallest filter sizes (LF 90/110 µm and CF 100 µm) yields the highest MFR values, indicating lower viscosity. Conversely, the larger LF size (110/130 µm) demonstrates relatively constant MFR values, ranging between 1.1 and 1.05 g/10 min, irrespective of CF variations. Especially, MFR values at a larger LF size tend to be smaller than those associated with a smaller LF. These observations align with the literature research [38,39], which suggests a correlation between particle size and a fluid’s viscous behavior. The literature indicates that, depending on the production, shape, and measurement methods, the viscosity of the chosen fluid either increases or decreases with increasing particle size.

In Figure 3b, the results of ash content measurements are depicted, revealing insights into the number of inorganic components. The smaller LF size (90/110 µm) in conjunction with the smallest CF size (100 µm) yields the lowest inorganic content, approximately 1.7%. As the CF size increases, a corresponding rise in inorganic substances is observed, reaching 2.4% at a CF size of 125 µm. The absence of one filter results in the highest inorganic content, peaking at 2.7%. The ash content of the larger LF size (110/130 µm) remains relatively constant at around 2.5%, with negligible deviations owing to their minor impact on the overall weight. As a point of interest, the ash content of the input material for the entire process was measured, resulting in the highest value of 3.6%, aligning with expectations.

In Figure 3c, the results of the oxidation onset temperature (OOT) measurements are presented, offering insights into the thermal stability of the material. The data for the smallest LF size (90/110 µm) reveal a decreasing trend in the OOT with an increase in CF size, reaching its minimum at the single filtration step. Similar tendencies are observed for the larger LF size, albeit with slightly lower OOTs. The deviations in OOT values are minimal. It is crucial to note that the OOT is highly influenced by stabilizers present in the material, and the number of those stabilizers varies strongly due to the input heterogeneity [40]. The measured OOT values are relatively low considering the typical processing temperatures of polyethylene in the range of 190 to 250 °C [41]. The rather low OOT may be attributed to the presence of contaminants in the material, specifically, organic remnants in the recyclate. These remnants might lead to premature oxidation or combustion of the material, resulting in the observed lower OOT values.

**Figure 5:**
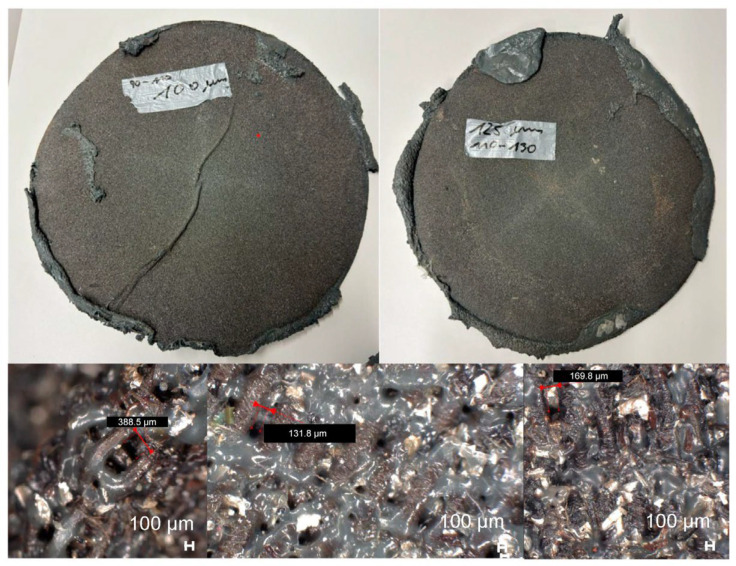
Picture of the CF with measured holes in the filter.

Figure 3d presents the results of optical contamination detection. As anticipated, a seemingly random distribution of contamination sizes is observed for particles smaller than 100 µm, consistent with the expectation that the filters were designed to capture particles starting from 100 µm. In specific detection regions (100–110 µm, 110–130 µm, and 130–200 µm), the combination of the smallest LF (90/110 µm) with the smallest CF (100 µm) yields the highest contamination amounts. Interestingly, pairing the same LF with CF 125 µm results in lower contamination numbers. The single filtration step consistently exhibits the lowest contamination levels. Contrasting behavior is observed with LF 110/130 µm; here, the combination with CF 100 µm produces the lowest contamination numbers, whereas CF 125 µm leads to higher values, and the single filtration step yields the highest contamination levels. Notably, larger particles (>200 µm) are prevalent across all samples. These observations align with phenomena identified in the literature research [42], where smaller screen sizes contribute to a faster and higher pressure build-up. This, in turn, results in the deformation of woven screens, enlarging their holes and increasing the contamination amounts inside the recyclate. The enlargement of the holes can be seen in Figure 5, which are microscopy pictures obtained using a Keyence VHX-7000 (Keyence International (Belgium) NV/SA, Mechelen, Belgium). Additionally, soft materials such as other polymers like polyethylene terephthalate (PET) or latex may deform and pass through the screens, further explaining the detected contamination trends. Furthermore, agglomeration after the second filtration cannot be ruled out. The total number of contaminations is listed in Table 3.

## 4. Conclusions

The findings indicate a notable impact of the filtration step on the processing behavior of the material. The observed correlation between the melt flow rate (MFR) values and filter sizes suggests potential influences from material decomposition or variations in particle sizes within the material. For clarification, further analysis such as gel permeation chromatography (GPC) or rheometrical analysis could be performed. Typically, polyethylene (PE) is processed within a temperature range of 190 to 250 °C. However, the results of the oxidation onset temperature (OOT) measurement emphasize the importance of cautious temperature selection. Opting for an excessively high temperature may contribute to premature degradation occurring within the extruder, underscoring the need for precision in temperature control to ensure optimal processing conditions. Moreover, post-stabilization to enable processing at higher temperatures could be another solution.

Melt filter systems exhibit greater effectiveness with “rigid” contaminations such as glass and metals, consistent with the observed increase in ash content with larger cartridge filter (CF) sizes. This correlation suggests that the filtration process efficiently captures inorganic components, contributing to a higher ash content. Conversely, the optical contamination detection reveals that organic components, characterized as “softer”, have a propensity to deform more readily, allowing them to bypass filtration screens. Despite this, the effectiveness of filtration is evident from the visible residue on the filter screens. The selection of the optimal filter combination and size depends on several factors, including the desired properties of the final product, the composition of the input stream in the overall process, and the economic constraints. In many instances, a single filtration step proves entirely sufficient, underscoring the importance of tailoring filtration strategies to specific needs and resource considerations. This nuanced understanding is pivotal for achieving an effective balance between product quality, process efficiency, and economic viability in recycling applications.

## Figures and Tables

**Table 1:** Filter sizes and different combinations of laser filter (LF) and cartridge filter (CF).

LF [µm]	CF [µm]
90–110	100
90–110	125
90–110	X
110–130	100
110–130	125
110–130	X

**Table 2:** Temperature profile of the recycling machine.

Feed Zone	Zone 1	Zone 2	Zone 3	Zone 4	Zone 5	Zone 6	Zone 7	Zone 8	Zone 9	Zone 10
100 °C	210 °C	215 °C	230 °C	235 °C	235 °C	215 °C	205 °C	205 °C	200 °C	200 °C

**Table 3:** Total number of contaminations in ppm.

	LF 90–110 CF 100	LF 90–110 CF 125	LF 90–110 CF X	LF 110–130 CF 100	LF 110–130 CF 125	LF 110–130 CF X	Sum
<100 µm	218,000	244,000	229,000	200,000	204,000	196,000	1,291,000
100–110 µm	20,000	19,000	18,000	17,000	17,000	17,000	108,000
110–130 µm	50,000	46,000	42,000	40,000	43,000	43,000	264,000
130–200 µm	166,000	157,000	139,000	128,000	136,000	137,000	863,000
>200 µm	546,000	534,000	572,000	615,000	600,000	607,000	3,474,000
Sum	1,000,000	1,000,000	1,000,000	1,000,000	1,000,000	1,000,000	

## Data Availability

The original contributions presented in the study are included in the article. Further inquiries can be directed to the corresponding author.
